# Robust and interpretable unit level causal inference in neural networks for pediatric myopia

**DOI:** 10.1038/s41746-026-02442-7

**Published:** 2026-02-19

**Authors:** Zihui Jin, Mengtian Kang, Wuyan Zhao, Wenjin Gui, He Li, Yongfang Tu, Yongjun Huo, Canqing Yu, Weihua Song, Ningli Wang, Xu Yang, Shi-Ming Li

**Affiliations:** 1 AETAS Lab.,School of Computer Science and Technology, Beijing Institute of Technology, Beijing, China; 2 Beijing Tongren Eye Center, Beijing Tongren Hospital, Beijing Institute of Ophthalmology, Beijing Key Laboratory of Intelligent Diagnosis Technology and Equipment for Optic Nerve-Related Eye Diseases, Capital Medical University, Beijing, China; 3 National Engineering Research Center for Ophthalmology, Beijing, China; 4 Engineering Research Center of Ophthalmic Equipment and Materials, Ministry of Education, Beijing, China; 5 Anyang Eye Hospital, Henan, China; 6 Department of Epidemiology and Biostatistics, School of Public Health, Peking University Health Science Center, Beijing, China; 7 Key Laboratory of Epidemiology of Major Diseases (Peking University), Ministry of Education, Beijing, China; 8 Department of Neurology, Xuanwu hospital Capital Medical University, Beijing, China; 9 National Clinical Research Center for Geriatric Diseases, Beijing, China

**Keywords:** Computational biology and bioinformatics, Diseases, Health care, Mathematics and computing, Medical research

## Abstract

Understanding causal mechanisms in deep learning is essential for clinical adoption, where interpretability and reliability are critical. Most existing AI systems act as black boxes, limiting transparency in medicine. We propose a causal inference framework integrated into neural networks to assess the influence of individual features on predictions. Using a prospective pediatric ophthalmology cohort of over 3000 children with longitudinal follow-up, our method estimates direct and indirect causal effects through intervention. Applied to myopia progression in children, our approach not only achieved good performance but also identified clinically plausible causal pathways. Refutation experiments with multiple falsification strategies confirm the robustness and reliability of causal effects. The approach is model-agnostic and suitable for digital health interventions requiring explainability. By incorporating unit-level causal reasoning into deep learning, this work advances transparent and reliable AI systems aligned with the goals of precision medicine and equitable healthcare.

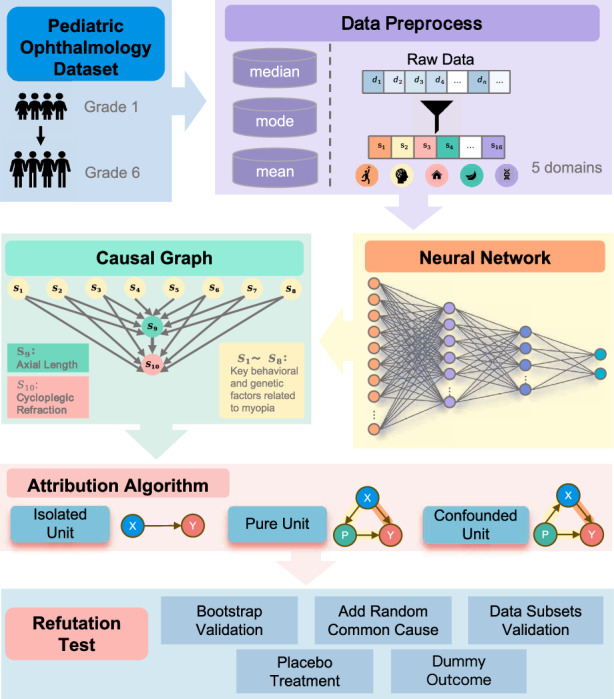

## Introduction

Myopia has become the most prevalent refractive disorder and a leading cause of vision impairment globally^1,2^. In East Asia, the adolescent myopia rate has reached alarming levels of 80–90%, with up to 20% developing high myopia—associated with sight-threatening complications, such as retinal detachment and macular degeneration^3–5^. The increasing incidence in younger populations calls for a deeper understanding of the multifactorial etiology of myopia^6–8^.

Traditional statistical methods, such as linear and logistic regression, have been widely used to explore myopia-related risk factors^9,10^. However, these models primarily capture correlations and often fail to represent the complex, nonlinear interactions inherent in biological systems^11–13^. Furthermore, few studies incorporate causal frameworks, limiting mechanistic insight and weakening the translational value for designing targeted interventions^14^.

With the rise of digital medicine, artificial neural networks (ANNs) offer an opportunity to leverage large-scale, real-world health data for predictive modeling^15–18^. Nevertheless, deep learning models are frequently criticized for their “black-box" nature, making it difficult to explain predictions or guide clinical action^19–23^. To address this limitation, post-hoc interpretability techniques, such as SHapley Additive exPlanations (SHAP), have been widely adopted to attribute model outputs to input features^24,25^. While such methods improve transparency, they primarily provide correlation-based explanations and do not fully capture the underlying causal mechanisms. Interpretability and reliability are particularly essential in medical settings, where clinical decisions must be transparent, justifiable, and safe.

To bridge this gap, there is growing interest in combining causal inference with neural architectures, enabling not only accurate predictions but also insight into the underlying mechanisms^26–29^. Such approaches are especially valuable for digital interventions, where understanding the impact pathways of behavioral, physiological, or environmental factors can inform scalable and personalized healthcare strategies.

In this study, we propose a causal modeling framework that integrates intervention-based causal attribution within deep neural networks. Using a prospective pediatric ophthalmology cohort tracking over 3000 children longitudinally, our framework disentangles both direct and indirect causal effects of input features on myopia progression, enhancing model transparency and clinical interpretability.

To achieve this, we categorize input neurons into three functional types—Isolated, Pure, and Confounded Units—based on their positions in the learned causal structure. We design targeted attribution strategies for each, including a domain-adaptive meta-learning approach for estimating causal effects under confounding bias. In addition to robust predictive performance, our model enables the reconstruction of interpretable causal pathways within the network, offering a mechanistic view of how input signals propagate through the architecture.

Importantly, our approach is model-agnostic and can be generalized to other clinical contexts that demand trustworthy, explainable AI. By integrating deep learning, causal reasoning, and real-world validation, our work contributes to the development of reliable digital interventions that meet the interpretive and ethical standards of modern healthcare.

## Results

### Neurons’ causal structure reveals functional categories of input neurons

**Fig. 1:**
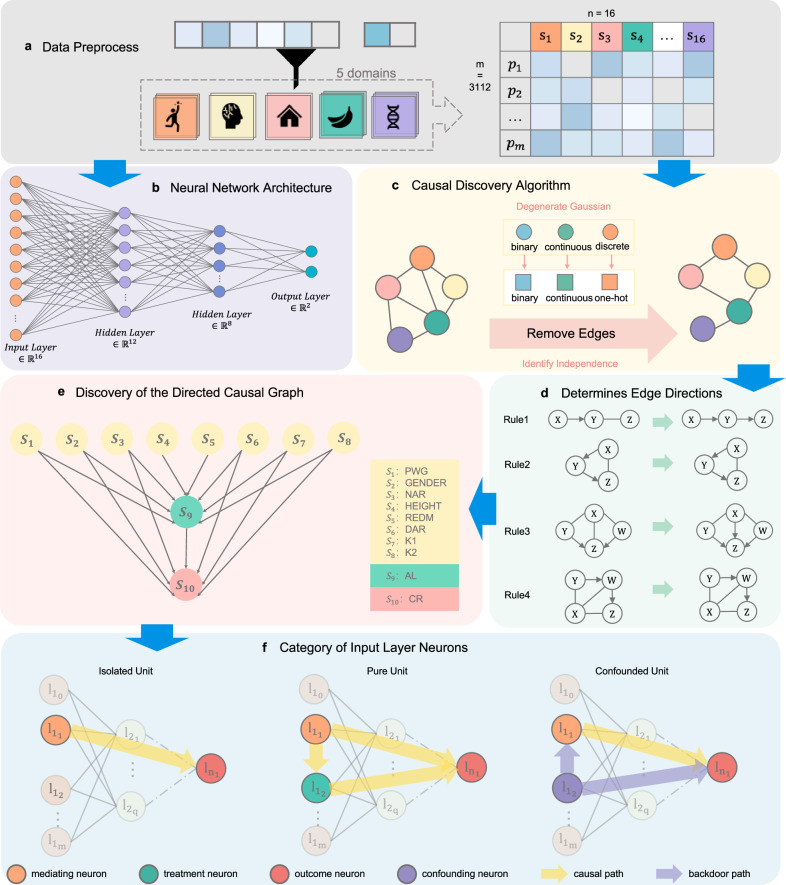
The workflow of causal structure discovery and functional categorization of input neurons. **a** Data preprocessing. Sixteen input variables were selected across five domains: behavioral, physiological, environmental, dietary, and hereditary. **b** Architecture of the neural network (NN). **c** Flow of removing edges via causal discovery algorithm. **d** Four rules for determining edge directions. **e** The directed causal graph of key behavioral and genetic factors related to myopia, involving 10 of the 16 input variables, connected by a total of 15 edges. **f** Three categorized units of the corresponding input-layer neurons in NN.

The causal explanation framework proposed in this study is model-agnostic, requiring that the prediction model be trained prior to causal analysis. We first constructed a binary classification model for myopia prediction using a feedforward neural network (as shown in Fig. 1b) trained on a pediatric ophthalmic cohort dataset collected via annual surveys in City Anyang, Henan Province, China, from 2011 to 2017 (see the section “Dataset”). As illustrated in Fig. 1a, the model input consists of 16 variables spanning behavioral, physiological, dietary, environmental, and genetic domains (Table 1). The result of this model for myopia classification is summarized in Table 2, the model achieved an accuracy of 0.933, indicating strong discriminative ability between the two classes. This result confirms that the binary myopia classification outcomes provide a robust foundation for subsequent causal attribution analyses, ensuring that the interpretability of the model is built upon clinically reliable predictions.

**Table 1:** Variables of the pediatric myopia dataset from the Anyang Childhood Eye Study

Variable	Variable meaning	Data type	Data range	Units
CR	Cycloplegic refraction	Continuous	−5.4 to 8.7	diopter (D)
AL	Axial length	Continuous	20–34	mm
NW	Total nearwork load	Continuous	0–102	hour (h)
DTO	Distance-viewing time outdoors	Continuous	0–57	hour (h)
NAR	Near accommodative ability of right eye	Continuous	−5.1 to 2.2	diopter (D)
DAR	Distance accommodative ability of right eye	Continuous	−6.6 to 4.4	diopter (D)
HEIGHT	Height	Continuous	97–143	cm
PULSE	Pulse rate	Continuous	52–140	Bpm
GENDER	Gender	Binary	0.1	0 represents a girl, 1 represents a boy
CB	Carbonated beverage consumption frequency	Discrete	1–5	times per week
EGG	Egg consumption frequency	Discrete	1–4	times per week
REDM	Red meat consumption frequency	Discrete	1–5	times per week
WHIM	White meat consumption frequency	Discrete	1–5	times per week
PWG	Number of parents wearing glasses	Discrete	0–2	person
K1	Corneal keratometry in both eyes	Continuous	38–71	mm
K2	Corneal keratometry in both eyes	Continuous	38–183	mm
Myopia	Myopia or not	Binary	0.1	0 represents non-myopia, 1 represents myopia

**Table 2:** Performance metrics of the four-layer feedforward neural network and traditional machine learning models

Model	Accuracy	Sensitivity	Specificity	F1 score	AUC
4-layer FNN	0.935 ± 0.011	0.939 ± 0.010	0.927 ± 0.019	0.947 ± 0.009	0.976 ± 0.007
SVM	0.863 ± 0.008	0.867 ± 0.013	0.854 ± 0.016	0.888 ± 0.007	0.921 ± 0.014
Logistic regression	0.935 ± 0.016	0.941 ± 0.009	0.926 ± 0.030	0.948 ± 0.013	0.977 ± 0.008
Naive Bayes	0.845 ± 0.018	0.783 ± 0.027	0.949 ± 0.011	0.864 ± 0.018	0.948 ± 0.010
*K*-Nearest neighbors	0.922 ± 0.012	0.936 ± 0.015	0.899 ± 0.011	0.938 ± 0.010	0.978 ± 0.008
Random forest	0.936 ± 0.014	0.940 ± 0.007	0.928 ± 0.027	0.948 ± 0.011	0.977 ± 0.009

To uncover the underlying causal relationships among input features, we applied a constraint-based causal discovery algorithm combining the PC algorithm^30^ with Degenerate Gaussian Scoring and dimensionality reduction via PCA. PCA was used solely as a preprocessing step to mitigate collinearity and improve numerical stability in conditional independence testing, while ensuring that the final causal graph is defined exclusively over the original 16 clinical and physiological variables (as shown in Fig. 1c, d). The final causal graph was constructed using the TETRAD software suite^31^. As shown in Fig. 1e, the resulting directed acyclic graph (DAG) reveals 15 causal edges connecting 10 of the 16 variables. Notably, six variables—parental myopia (PWG), gender (GENDER), near and distance accommodative ability (NAR, DAR), and corneal curvature (K1, K2)—were found to be direct causes of both axial length (AL) and cycloplegic refraction (CR). Height and red meat consumption (REDM) showed direct effects on AL only, while AL was identified as a direct cause of CR.

Based on Fig. 1f, we categorized the corresponding input-layer neurons in the trained neural network into three structurally distinct types: Isolated Units, Pure Units, and Confounded Units. This categorization is not merely descriptive; rather, it enables a principled decomposition of causal contributions within the neural network. By aligning unit types with distinct structural roles in the causal graph, we introduce a unified framework to estimate causal effects under different confounding conditions, thus bridging structural causal inference and neural attribution in a theoretically grounded manner.

Isolated Units (Fig. 1f, left one) are input neurons that do not have any causal relation with other input features. Their contributions to the prediction model are assumed to be independent. Six variables fall under this category: CB, EGG, NW, PULSE, WHIM, and DTO.

Pure Units (Fig. 1f, middle one) are input neurons that influence the output only through downstream mediators. For example, variable A causally influences variable B, which in turn affects the prediction output. This corresponds to a chain-like causal path. Variables such as PWG, GENDER, NAR, DAR, K1, K2, HEIGHT, and REDM are identified as Pure Units based on their positions in the causal graph.

Confounded Units (Fig. 1f, right one) are input neurons whose effect on the output is entangled with that of another causally related variable, forming a v-structure or a backdoor path. In our study, AL and CR are identified as Confounded Units, both influenced by multiple upstream variables and interlinked causally.

This categorization is central to the subsequent causal attribution analysis. By structurally disentangling how input neurons contribute to predictions, we establish a scalable and interpretable framework for assessing causal roles within neural networks in medical applications.

### Causal attribution experiments on isolated units reveal consistency and deviation from prior knowledge

We performed causal attribution experiments on the six Isolated Units identified in the causal graph to assess the individual causal effect of each input-layer neuron on the output predictions of the trained neural network. In this context, attribution refers specifically to estimating the average treatment effect (ATE) of a single input neuron on each output class in the binary classification task (myopic vs. non-myopic).

Each Isolated Unit was treated as an intervention variable and directly input into a causal attribution algorithm to quantify its effect. Since the model produced a binary prediction for myopia status (Yes/No), we conducted separate interventional analyses on both output classes.

**Fig. 2:**
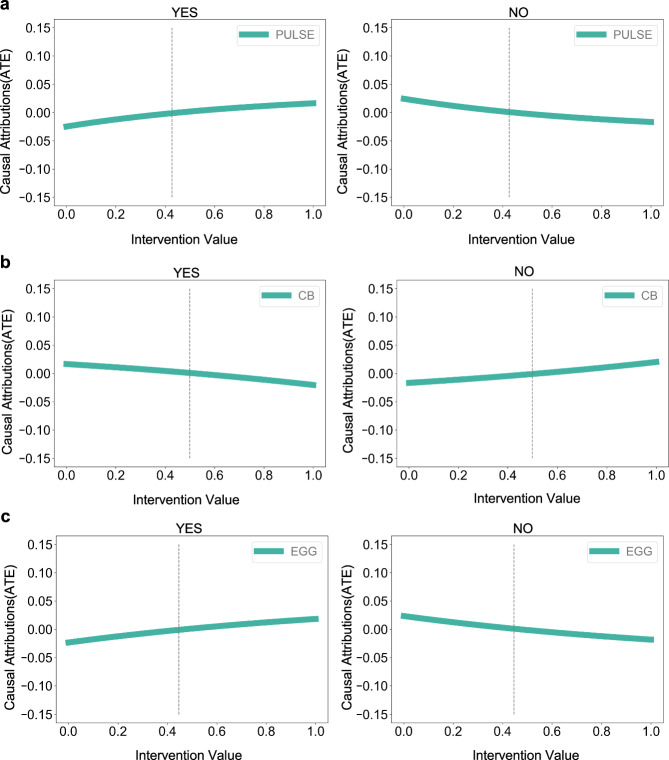
Causal attribution results of Isolated Units. **a** Intervention value of PULSE. **b** Intervention value of CB. **c** Intervention value of EGG. The difference between the ATE value and its average value is presented as the green line, which shows the increasing/decreasing trend of the variable for myopic. 'Yes' means myopic and 'No' means non-myopic. The vertical dotted line indicates that the ATE at this location is 0.

Pulse rate (PULSE) has been previously reported to be negatively associated with myopia risk^32–35^, possibly due to increased ocular blood flow influencing eye growth. As shown in Fig. 2a, increasing the intervention value of PULSE leads to a rising trend in the ’No’ (non-myopic) output neuron’s ATE and a decreasing trend in the ’Yes’ (myopic) output neuron’s ATE. This suggests a negative causal relationship between PULSE and myopia, in agreement with prior clinical findings and indicating that the model has accurately captured this relationship.

In contrast, carbonated beverage consumption frequency (CB) and egg consumption frequency (EGG) have been reported to be positively associated with myopia^36–39^. However, as shown in Fig. 2b, c, the model exhibits an inverted trend: CB shows a positive effect on the ’No’ neuron and a negative effect on the ’Yes’ neuron, while EGG shows the opposite pattern. These observations indicate that the model’s learned causal behavior for these features is inconsistent with established domain knowledge. A likely explanation is the uneven distribution of CB and EGG consumption across subgroups in our dataset, which may have influenced the model’s learned patterns.

In summary, among the six Isolated Units, the model correctly captures causal trends for PULSE and DTO, but misrepresents those for NW, CB, and EGG. The effect of WHIM appears negligible and is excluded from further interpretation^40–45^. Detailed attribution results of variables other than CB, EGG, and PULSE are shown in Supplementary material Figs. S1–S3.

### Causal attribution on pure units confirms model reliability across mediated paths

**Fig. 3:**
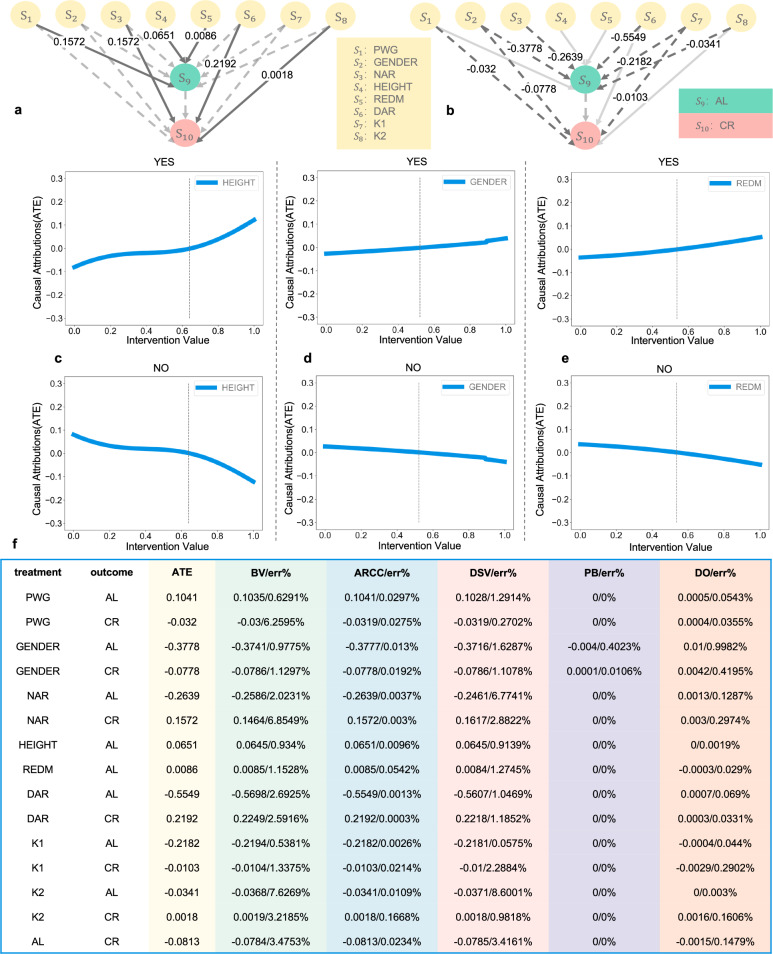
Causal attribution results of Pure Units. **a** Result of positive causal effects. **b** Result of negative causal effects. Solid lines represent positive causal effects and dashed lines indicate negative effects. Numbers on each line represent the causal effect value of the corresponding causal links. **c–e** Intervention value of HEIGHT, GENDER, and REDM. The difference between the ATE and its average value is presented as the blue line, showing the increasing/decreasing trend of variable for myopic. 'YES' means myopic and 'NO' means non-myopic. The vertical dotted line indicates that the ATE at this location is 0. **f** Refutation results of the 15 causal effects.

For Pure Units, causal attribution can be conducted without adjusting for additional covariates, as their causal effects are either direct or fully mediated by identified intermediate variables. The attribution process involves identifying all causal paths—both direct and indirect—between the input neuron and the output neuron. Assuming linear causal relationships among the variables in the dataset, we performed causal inference using linear regression applied to the structure encoded in the causal graph. The resulting causal effect graph is shown in Fig. 3a, b, where solid lines represent positive causal effects and dashed lines indicate negative effects.

Two representative examples are shown in Fig. 3c, d, where we analyze the effect of height (HEIGHT) and gender (GENDER). As the intervention value of HEIGHT increases, the response of the ’No’ (non-myopic) output neuron shows a decreasing trend, while the ’Yes’ (myopic) output neuron shows an increasing trend. This trend is consistent with previous research findings that people with greater height are more likely to suffer from myopia^46–52^. It should be emphasized, however, that the causal pathway identified by our framework is HEIGHT → AL → CR, rather than a direct HEIGHT → Myopia link. This indicates that height acts indirectly through ocular growth and refractive status, and should not be interpreted as a direct or modifiable risk factor for myopia. The trend after the intervention on gender (GENDER) is the same as previous findings, that is, among adolescents, the incidence of myopia in females is higher than that in males^53–60^. However, such associations may vary with individual-level heterogeneity.

Among the eight Pure Units, the model correctly reflects the causal trends for NAR, DAR, K1, K2, HEIGHT, GENDER, and PWG^61–89^. Notably, while axial length (AL) is widely recognized as the primary anatomical determinant of myopia and a direct driver of cycloplegic refraction (CR), several clinical studies have reported that accommodative function—including near and distance accommodative ability (NAR and DAR)—may also contribute to AL elongation and myopia progression^90–93^. Therefore, the NAR/DAR → AL path revealed by our framework should be interpreted as a data-driven attribution pattern that aligns with certain clinical observations, while requiring further validation. Detailed attribution results of variables other than HEIGHT and GENDER are shown in Supplementary material Figs. S4–S7. The effect of REDM remains inconclusive in the existing literature and is therefore not interpreted in terms of fit correctness. However, our method revealed a weak but directional causal path from REDM to AL (as shown in Fig. 3b *S*_5_ → *S*_9_), followed by a stronger negative causal influence from AL to CR (as shown in Fig. 3b *S*_9_ → *S*_10_). Although the direct effect of REDM on AL is marginal, as shown in Fig. 3e, the existence of this indirect pathway suggests a potential mediating mechanism by which dietary patterns might influence refractive development. This finding, uncovered through our causal attribution framework, suggests a hypothesis-generating signal that warrants further investigation in nutritional ophthalmology and longitudinal dietary studies.

### Meta-learning-based attribution for confounded units demonstrates stable causal estimation

To robustly assess causal effects under confounding, especially in cases where input neurons share common ancestors with output nodes, conventional regression-based methods often yield biased estimates. To address this challenge, we introduce a domain-adaptive meta-learning algorithm that leverages covariate balancing through propensity scores. The key idea is to learn a causal representation that generalizes across input distributions, enabling unbiased estimation even when confounders are present. This framework not only corrects for selection bias but also enhances robustness under covariate shift—making it well-suited for causal inference in complex medical prediction tasks. Given that these input neurons are causally entangled with others and subject to confounding with the output layer, we first identified and controlled for confounding variables using the backdoor criterion, enabling the estimation of unbiased causal effects.

To evaluate the performance of different causal effect estimation models, we adopt the R score metric based on the R-Learner framework. The R score is defined as

1 $${\rm{R}}\,{\rm{score}}=1-\frac{{\hat{L}}_{{\rm{R}}}}{{L}_{{\rm{base}}}}$$

where ${\hat{L}}_{{\rm{R}}}$ is the R-loss obtained from cross-validation using the estimated treatment effect model $\widehat{\tau }(x)$, and *L*_base_ is the baseline loss computed using a constant average treatment effect.

This metric assesses the ability of the estimated individualized treatment effect to explain residual variation, in comparison to a constant effect. A higher R score indicates better performance in modeling heterogeneous treatment effects. Our proposed meta-learning model achieved superior accuracy, with the best result obtained using a GBDT-based estimator (R score = 0.3850). Further improvements were achieved via an ensemble method combining GBDT and Random Forest with a 10:1 weight ratio, yielding an R score of 0.6277, indicating strong attribution performance (see Supplementary material for details).

**Fig. 4:**
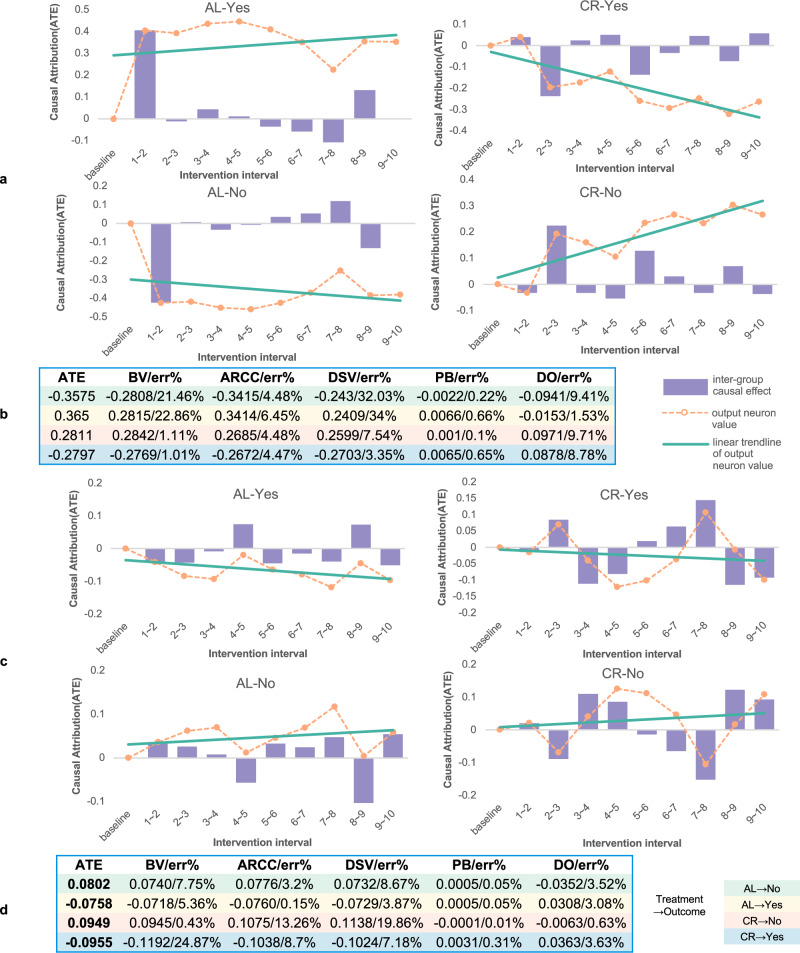
Causal attribution results of Confounded Units. **a** and **c** Causal effects of AL and CR on output neurons (Yes/No) under equal-width and equal-frequency discretization, respectively. Equal-width discretization divides AL and CR into 10 groups within their value intervals and calculates the ACE between adjacent discrete groups. The equal-frequency discretization divides variables into 10 groups, and the number of data points in each group is the same. The output neuron value (Yes/No) was benchmarked at 0. The corresponding causal effect value was added to each intervention interval, from which the dashed line of the output neuron value change showed. **b** and **d** Refutation results of equal-width and equal-frequency discretization, respectively.

To mitigate bias introduced by discretizing continuous variables, we applied both equal-width and equal-frequency discretization strategies to the two Confounded Units—axial length (AL) and cycloplegic refraction (CR)—and estimated their average causal effects (ACE) on the model’s binary outputs (’No’ and ’Yes’). As shown in Fig. 4a, c, AL exhibits a positive causal effect on myopia risk, consistent with established clinical findings^94–96^. Similarly, CR shows a pattern where lower refractive power corresponds to increased myopia risk^97–99^. Equal-width discretization provides a clearer causal trend compared to equal-frequency discretization.

Importantly, the causal attribution trends for both AL and CR are aligned with current medical understanding, indicating that the model correctly internalized known relationships between these variables and myopia.

### Refutation experiments validate causal robustness

To evaluate the internal validity and robustness of our estimated causal effects, we conducted a series of refutation experiments inspired by the DoWhy framework^100^. These experiments were designed to determine whether our results hold under systematic perturbations to data and model assumptions.

Five types of intervention strategies were employed: bootstrap resampling, random addition of artificial confounders, subset validation, placebo treatment, and dummy outcome substitution.

The results of the Pure Units refutation experiment are shown in Fig. 3f. It can be seen from the figure that the error rates (err%) obtained in the five refutation experiments are all <10%, and there is no situation where the error rates of the five refutation experiments are all >1%.

The results of the Confounded Units refutation experiment are shown in Fig. 4b, d. For AL, equal-frequency discretization produced lower refutation error, while CR showed greater stability with equal-width discretization. These results collectively demonstrate that the model is capable of producing accurate and stable causal attributions even under confounding conditions.

## Disscusion

In this study, we present a causal learning-based interpretability framework for neural networks, tailored to the clinical prediction of pediatric myopia. By abstracting a feedforward neural network into a structured causal system, we quantify the causal contribution of each input neuron to the model output and implement targeted attribution strategies according to the neuron’s structural role in the learned causal graph. Using a longitudinal dataset collected in City Anyang, Henan Province, China (2011–2017), our method integrates causal discovery, mediation analysis, and domain-adaptive estimation to produce interpretable and robust neural reasoning.

The inferred causal graph, constructed using a constraint-based algorithm (PC with Degenerate Gaussian Scoring), revealed stable directed dependencies among the 16 input features. Based on this structure, we categorized input neurons into three functionally distinct types—Isolated, Pure, and Confounded Units—each representing a unique causal configuration. Attribution results for Isolated Units aligned with established risk factors in several cases: for instance, increased pulse rate and outdoor activity were found to have protective effects against myopia, consistent with previous studies^101^. In contrast, misalignment was observed for some dietary variables (e.g., carbonated beverage and egg consumption), suggesting either data limitations or prior model biases. Pure Units, evaluated through linear path-based inference, demonstrated biologically plausible effects—e.g., the positive causal influence of height on myopia risk was found to be mediated through axial elongation. For Confounded Units such as axial length (AL) and cycloplegic refraction (CR), our meta-learning-based estimation—guided by the backdoor criterion—produced coherent and stable causal effects. This was further validated by a suite of counterfactual perturbation experiments.

These results demonstrate that black-box neural networks can be decomposed into biologically meaningful, causally interpretable substructures^102,103^. The model’s ability to internalize known physiological mechanisms, particularly around AL and CR—which directly modulate the eye’s focal characteristics—confirms its biological plausibility. Overall, the causal pathways identified by our framework demonstrate strong biological plausibility and align closely with established clinical knowledge. Throughout the study, we placed particular emphasis on clinical interpretability, and for each major result, we cross-referenced supporting ophthalmic literature to ensure consistency with prior findings. We fully acknowledge the central role of axial length (AL) as the dominant anatomical determinant of myopia, and our model’s detection of the AL → CR relationship is fully consistent with this consensus. At the same time, our framework is inherently data-driven: the causal paths it reveals should be understood as internal attribution patterns within the predictive model, which require cautious interpretation in light of existing clinical evidence. Notably, we found that many of these data-driven paths are corroborated by previous studies, reinforcing the clinical validity of our approach. Compared to conventional attribution methods like SHAP or LIME^104–108^, which often fail under confounding or produce unstable saliency maps, our causal framework ensures interpretability by explicitly modeling conditional dependencies and causal paths. This not only improves explanation reliability but also enables insight into systemic bias or misattribution patterns—critical for deploying AI safely in clinical settings.

Importantly, our method has direct implications for the design of digital interventions and early prevention strategies. The identification of modifiable behavioral risk factors, such as outdoor activity, as causal precursors to myopia progression suggests that individualized lifestyle adjustments could be guided by our model outputs. Additionally, we identify discrepancies between learned model logic and medical consensus, which could be used to iteratively improve neural architectures or training protocols, enhancing both performance and clinical acceptability.

At the same time, we note that several features exhibited attribution patterns that diverged from previously established clinical or epidemiological knowledge. These discrepancies may, at least in part, arise from distributional characteristics of the dataset—such as uneven representation of certain behaviors across age or socioeconomic subgroups—which can shape the model’s learned associations. It is important to emphasize that our causal framework is primarily intended to disentangle and visualize the internal attribution logic of predictive models, rather than to fully correct for population-level confounding. Consequently, while the framework enhances interpretability and highlights biologically plausible pathways, residual distributional imbalances may still produce effects inconsistent with consensus knowledge. We regard this as a constructive signal, since such mismatches can reveal potential data artifacts or, alternatively, point to novel hypotheses warranting further validation.

Despite these promising findings, several limitations remain. First, the accuracy of the causal discovery process relies on the completeness and quality of observational data. In particular, the presence of missing covariates or unobserved confounders may bias the inferred graph structure. Furthermore, our method was validated on complete longitudinal data, which may not fully reflect real-world scenarios where data are often incomplete or irregularly sampled. Using complete cases may introduce selection bias if missingness is not completely at random. To address incomplete data in future applications, our framework could be extended through multiple imputation techniques for missing values before causal discovery, or through modified causal discovery algorithms that handle missing data directly. Second, our mediation model currently assumes linear causal effects, while robustness is verified through counterfactual tests, highly nonlinear interactions may not be fully captured. Kernel-based or neural causal inference methods may be needed to improve generalizability. Third, while this study chose a feedforward architecture, our framework is designed to be model-agnostic due to the decoupling of causal discovery from neural attribution. In practice, extending this method to other architectures would primarily involve substituting the attribution module while maintaining the structural causal categorization logic. For instance, when applying the framework to convolutional neural networks (CNNs) for retinal imaging, input units could be defined as high-level features extracted from specific regions of interest, and attribution could be estimated via Grad-CAM or Integrated Gradients. For transformer-based models handling genomic sequences, the nodes could represent token embeddings or attention heads, utilizing Attention Rollout or perturbation-based attribution to quantify causal contributions.

Looking ahead, our work opens several directions for innovation: (1) Embedding causal graphs into neural network training could lead to structure-aware architectures that align model connectivity with known biomedical pathways. (2) Extending our framework to cross-modal causal interpretation—bridging electronic health records with imaging or sensor data—would further improve clinical relevance. Such an extension would leverage the framework’s modularity, where the universal “causal skeleton" derived from data governs the interpretation of diverse neural components across different sub-networks. (3) As causal attributions can highlight mislearned associations, they may serve as real-time monitors for algorithmic fairness and bias. (4) With temporally dense datasets, our framework can evolve into dynamic causal modeling, enabling the simulation of patient-specific interventions for myopia prevention or progression control.

In conclusion, we introduce a rigorous and generalizable framework for unit-level causal attribution in neural networks, validated in a clinically relevant setting. By focusing on the interpretation of the model in causal principles, we offer a pathway toward more transparent, robust, and trustworthy AI systems for digital medicine.

## Methods

### Dataset

We used the Dataset of a prospective longitudinal school-based cohort study—the Anyang Childhood Eye Study (ACES)^109^, one of the largest longitudinal studies of myopia in school-aged children in China. The dataset was collected through annual follow-up surveys from 2011 to 2017 and included 3112 first-grade students at baseline, who were followed through sixth grade (school grades 1–6, spanning six academic years). Each year corresponded to a single visit per participant, with examinations conducted once annually at their schools. At baseline, participants had a mean age of 7.1 years (range: 5.7–9.3 years), and 57.8% were male. Data collection was conducted in collaboration with local primary schools under the supervision of certified medical professionals and epidemiologists. The study received ethics approval from the Institutional Review Board of Beijing Tongren Hospital, Capital Medical University, and informed consent was obtained from all participants and their guardians. Written informed consent was obtained from all participants and their parents or legal guardians.

All children provided a written informed consent form signed by their parents, and verbal consent was also obtained from each child. This study adhered to the tenets of the Declaration of Helsinki. Ethics committee approval was obtained from the Institutional Review Board of Beijing Tongren Hospital, Capital Medical University (TRECKY2018-030).

The survey captured a broad range of data across behavioral, physiological, environmental, dietary, and hereditary domains. After preprocessing, 2748 records with complete and valid longitudinal data were retained for analysis (88.3% of baseline participants). These records spanned a 6-year follow-up period, enabling the construction of a rich temporal feature space suitable for causal discovery and model training. In this survey, myopia was classified as a binary outcome (0 = non-myopia, 1 = myopia) according to cycloplegic refraction (CR > −0.5 D for non-myopia, CR ≤ −0.5 D for myopia). The myopia classification was determined based on whether the child developed myopia at any point during the 6-year follow-up period, rather than solely at the last visit, thereby capturing incident myopia cases throughout the study period.

To minimize potential methodological bias, different preprocessing strategies were applied depending on the temporal characteristics of each variable. For dynamic ophthalmic measures (e.g., CR), we derived annual progression rates rather than simple averages. For behavioral and dietary variables (e.g., NW, DTO, CB, EGG, REDM, WHIM), cumulative values across 6 years were used to capture long-term exposure. For relatively stable biometric and physiological measures (e.g., NAR, DAR, PULSE, AL, K1, K2), 6-year averages were computed to reduce random fluctuations and measurement error. Static variables such as gender and parental myopia (PWG) were retained as baseline values.

After data preprocessing (see Supplementary material for details), 16 variables were finally selected and used as input for model training and causal attribution. A full list of selected variables and their definitions is provided in Table 1.

### Neural network architecture

We constructed a feedforward neural network (FNN) model to predict myopia status based on the preprocessed features derived from the dataset. The network architecture consists of an input layer with 16 neurons corresponding to the selected variables, two fully connected hidden layers with 12 and 8 neurons, respectively, and an output layer with 2 neurons representing binary classification outcomes (myopia vs. non-myopia). The network structure is illustrated in Fig. 1b.

Each neuron in the hidden layers uses the Rectified Linear Unit (ReLU) as the activation function to introduce non-linearity. The final output layer employs the softmax function to produce probability distributions over the two classes. The model is trained using the cross-entropy loss function and optimized via stochastic gradient descent with backpropagation.

To identify optimal model performance, we conducted a series of hyperparameter tuning experiments, adjusting the learning rate, batch size, and number of training epochs. The best-performing configuration was selected based on validation accuracy. Final performance metrics of the four-layer FNN model on the test set are reported in Table 2, demonstrating high accuracy and robustness across multiple evaluation criteria.

To benchmark performance, we also implemented five traditional machine learning models—logistic regression, Naive Bayes, random forest, *K*-nearest neighbors, and support vector machine—using the same input features. As shown in Table 2, the 4-layer FNN achieved consistently high performance across all evaluation metrics. Its sensitivity was comparable to that of SVM and logistic regression, all of which reached the highest values among the tested models (within the allowable statistical error), thereby reducing the risk of missed myopia cases. Compared with other baseline models, the FNN also provided a more balanced trade-off between sensitivity and specificity, indicating its robustness in distinguishing myopia versus non-myopia. Furthermore, given its representative “black-box” nature, the FNN serves as an appropriate foundation for our proposed causal explanation framework, which aims to move beyond predictive accuracy to provide interpretable and clinically meaningful insights.

### Causal discovery

Causal discovery, a key approach for inferring causal relationships from observational data via graphical models like DAGs, enables researchers to uncover intervention-relevant structures in the absence of randomized trials^110^. In this study, we adopted a constraint-based causal discovery approach, specifically the Peter-Clark (PC) algorithm^30^, to identify causal relationships among the 16 input variables of the myopia prediction model. The PC algorithm begins with a fully connected undirected graph and iteratively removes edges by testing conditional independence. It then determines edge directions using v-structure identification and Meek rules^111^, producing a partially directed acyclic graph (CPDAG) that represents the underlying causal structure.

A critical challenge in biomedical datasets is the presence of mixed-type variables, including both continuous and categorical features. Traditional independence tests (e.g., Pearson correlation, Chi-squared test, or Fisher’s *Z*-test) are limited to continuous or discrete data and often fail in mixed domains.

To address this, we integrated the degenerate Gaussian likelihood ratio test (DG-LRT) into the PC algorithm. This method, originally proposed by Andrews^112,113^ and refined for mixed data, uses a transformed representation of categorical variables via one-hot encoding, followed by degenerate Gaussian modeling. The resulting structure is especially well-suited for medical applications where variable types and scales vary widely.

This design highlights the cross-disciplinary adaptability. It is capable of capturing statistically reasonable causal structures across hybrid domains, bridging data mining, statistical learning, and clinical reasoning.

For stability and completeness, the causal discovery process was conducted on the entire dataset rather than a training split, ensuring that the learned structure reflected global dependencies among variables.

The final causal graph extracted from our dataset included 10 out of the 16 input variables, forming 15 directed edges (as shown in Fig. 1e). This structure serves as the backbone for the following causal attribution and interpretation. The detailed steps of the algorithm are presented in Algorithm 1.

#### Algorithm 1

PC algorithm with a degenerate Gaussian likelihood ratio test 

### Attribution algorithm

In this study, causal inference is conducted at two levels: estimating the effect of input-layer neurons on output neurons within a neural network (i.e., attribution) and evaluating inter-feature causal effects in the dataset using regression-based techniques.

Causal effects can be defined at different levels. Assuming binary treatment *T* ∈ {0, 1}, the most commonly used is

Average treatment effect (ATE):

$$\,{\rm{ATE}}\,={\mathbb{E}}[Y(1)]-{\mathbb{E}}[Y(0)]$$

Isolated Units: Isolated Units refer to specific input neurons in a neural network whose causal effects on output neurons are to be independently assessed. Based on the operator framework introduced in Section *Causal Inference*, we formalize the average causal effect (ACE) from input neuron *x*_*i*_ to output neuron *y*, as shown in Fig. 1f left one.

For a binary variable *x*, its ACE on another variable *y* is defined as

2 $${\rm{ACE}}(x)={\mathbb{E}}[y| {\rm{do}}(x=1)]-{\mathbb{E}}[y| {\rm{do}}(x=0)]$$

For continuous-valued variables, we extend this to

3 $${{\mathrm{ACE}}}_{{x}_{i}}=\frac{1}{{high}^{i}-{low}^{i}}{\int }_{{low}^{i}}^{{high}^{i}}{\mathbb{E}}[y|{\mathrm{do}}({x}_{i}=\alpha )]\,d\alpha$$

To mitigate the inefficiency and variance introduced by high-dimensional sampling, we approximate the intervention expectation using a second-order Taylor expansion of the network function *f*.

Let *μ* be the mean input vector, and *f*(*μ*) be the smooth neural network function. For a small perturbation *δ* in the input, the Taylor expansion around *μ* is

4 $$f(\mu +\delta )\approx f(\mu )+\nabla f{(\mu )}^{\top }\delta +\frac{1}{2}{\delta }^{\top }{\nabla }^{2}f(\mu )\delta$$

Taking expectation over *δ* where ${\mathbb{E}}[\delta ]=0$ leads to:

5 $${\mathbb{E}}[y|{\mathrm{do}}({x}_{i}=\alpha )]\approx f(\mu )+\frac{1}{2}\,{\mathrm{tr}}({\nabla }^{2}f(\mu )\cdot \mathrm{Cov})$$

This allows efficient estimation of intervention effects without full re-evaluation over the dataset.

The algorithm estimates the causal effect of input neuron *x*_*i*_ on the output neuron *y* over a specified intervention interval [low^*i*^, high^*i*^] by dividing it into num uniform sub-intervals.

The full procedure is shown in Algorithm 2.

#### Algorithm 2

Approximate causal effect for isolated input unit 

*Pure Units*: This section discusses how to attribute causal effects for input neurons that are causally dependent on other inputs but are not affected by confounders—referred to as Pure Units. We assume these neurons are connected to other input neurons via causal pathways, but no common ancestor influences both the input and output neurons (i.e., no confounding exists).

As illustrated in Fig. 1f, middle one, let *l*_11_ be the intervened input neuron and *l*_*n*1_ be the output neuron. In this structure, *l*_11_ can influence *l*_*n*1_ through multiple paths, such as the direct path *P*_1_ and an indirect path *P*_2_ via intermediate neuron *l*_12_, but no confounders are present.

In this scenario, the causal effect of *l*_11_ on *l*_*n*1_ includes both direct and indirect components:

6 $${\rm{ACE}}({l}_{11}\to {l}_{n1})={{\rm{ACE}}}_{{P}_{1}}+{{\rm{ACE}}}_{{P}_{2}}+\cdots$$

Each term corresponds to a causal path, and the effect along each path can be estimated as the product of edge weights along that path. For instance, the average causal effect along the indirect path *P*_2_ from *l*_11_ → *l*_12_ → *l*_*n*1_ is given by

7 $${{\rm{ACE}}}_{{P}_{2}}=\left({\mathbb{E}}[{l}_{12}| {\rm{do}}({l}_{11}={\alpha }_{2})]-{\mathbb{E}}[{l}_{12}| {\rm{do}}({l}_{11}={\alpha }_{1})]\right)\cdot {\beta }_{{l}_{n1} \sim {l}_{12}}$$

where ${\beta }_{{l}_{n1} \sim {l}_{12}}$ denotes the regression coefficient from *l*_12_ to *l*_*n*1_. Equation (7) reflects the mediated effect of *l*_11_ on *l*_*n*1_ through *l*_12_. The direct effect ${\beta }_{{l}_{n1} \sim {l}_{11}}$ accounts for path *P*_1_.

Once all relevant paths are identified and estimated, we update the mean vector *μ* and covariance matrix Σ of the training data to reflect the intervened distribution. The expected effect of *l*_11_ on *l*_*n*1_ under intervention do (*l*_11_ = *α*) is then computed using the intervention expectation approximation from the previous subsection:

8 $${\mathbb{E}}[{l}_{n1}|{\mathrm{do}}({l}_{11}=\alpha )]\approx f(\mu )+\frac{1}{2}\cdot {\mathrm{tr}}({\nabla }^{2}f(\mu )\cdot \Sigma )$$

This method effectively aggregates the impact of multiple causal paths while maintaining computational efficiency. By correcting for potential bias induced by intermediaries, it enables robust causal interpretability in neural networks.

The full procedure is shown in Algorithm 3.

#### Algorithm 3

Estimate causal effect for pure input unit 

*Confounded Units*: In real-world neural networks, causal dependencies among input neurons are often interleaved with hidden confounding variables. A confounder is a variable that simultaneously influences both the intervention (input neuron) and the outcome (output neuron), thereby creating backdoor paths that induce selection bias and invalidate naive causal estimation.

As shown in Fig. 1f, right one, the input neuron *l*_11_ affects output neuron *l*_*n*1_ via the direct causal path *P*_1_: *l*_11_ → *l*_*n*1_. However, the presence of neuron *l*_12_, which also influences *l*_*n*1_ and is influenced by *l*_11_, forms a backdoor path *l*_11_ ← *l*_12_ → *l*_*n*1_. This makes *l*_12_ a confounder and it needs to be adjusted to avoid biased estimation of the average treatment effect (ATE) of *l*_11_ on *l*_*n*1_.

To estimate unbiased causal effects in the presence of confounders, we adopt a four-stage framework that integrates graphical causal identification, propensity-based domain adaptation, and ensemble-based meta-learning^114^. Specifically:

*Confounder identification*: Based on a predefined causal graph, we identify a sufficient set of covariates *Z* that satisfy the backdoor criterion, thereby blocking all spurious associations between the treatment variable *X* and the outcome *Y*.

*Propensity score estimation*: We estimate the propensity score *e*(*z*) = *P*(*X* = 1∣*Z* = *z*) using a supervised learning model. These scores are subsequently used to reweight samples and mitigate distributional imbalance across treatment groups, enabling domain adaptation.

Ensemble meta-learning for outcome modeling: To model potential outcomes, we train ensemble-based predictors using gradient boosted decision trees (GBDT) and random forests (RF), combined with a fixed weight ratio of 10:1. These models are trained separately on the treatment and control groups with inverse-propensity weighting to account for confounding.

*Causal effect estimation*: Individual treatment effects (ITEs) are approximated by computing residuals between observed and predicted counterfactual outcomes. A final regression model is fitted to these residuals to estimate the conditional average treatment effect $\widehat{\tau }(x)$. Prior to modeling, continuous covariates such as axial length (AL) and cycloplegic refraction (CR) are discretized using both equal-width and equal-frequency discretization to satisfy the discrete input requirements of the meta-learning model.

#### Algorithm 4

Domain-adaptive causal attribution via ensemble-based meta-learning 

### Refutation experiments

To evaluate the robustness and reliability of the estimated causal effects, we conduct a series of refutation experiments following the methodology proposed in DoWhy^100^. These experiments are designed to examine whether the estimated effect is stable under a variety of controlled perturbations. Specifically, five types of falsification strategies are applied:

*Bootstrap validation (BV)*: This method uses resampling with replacement from the original dataset to generate synthetic bootstrap datasets. A reliable causal effect estimator should produce consistent estimates across these samples. The error rate is defined as

9 $${{E}{R}{R}}_{BV}=\left|\frac{New\,Effect-Estimated\,Effect}{Estimated\,Effect}\right|$$

*Add random common cause (ARCC)*: This method randomly introduces artificial confounding variables into the dataset. A robust estimator should show minimal change in causal effect when noise variables are added. The deviation indicates sensitivity to confounding bias. The error rate is calculated as

10 $${{E}{R}{R}}_{ARCC}=\left|\frac{New\,Effect-Estimated\,Effect}{Estimated\,Effect}\right|$$

*Data subsets validation (DSV)*: This method randomly selects a subset of the data as the new evaluation set and recomputes the causal effect. A stable estimation algorithm should yield similar results across data splits. The error rate is defined as

11 $${{E}{R}{R}}_{DSV}=\left|\frac{New\,Effect-Estimated\,Effect}{Estimated\,Effect}\right|$$

*Placebo treatment (PT)*: In this test, the original treatment variable is replaced by a randomly permuted variable (i.e., placebo). Since there is no actual treatment effect, a valid causal estimator should report an effect close to zero. The error rate is defined as

12 $${{E}{R}{R}}_{PT}=\left|New\,Effect\right|$$

*Dummy outcome (DO)*: In this setting, the outcome variable *Y* is replaced with a randomly generated variable. If the causal inference method is valid, it should yield an estimated causal effect near zero. The error rate is

13 $${{E}{R}{R}}_{{D}{O}}=\left|New\,Effect\right|$$

These falsification strategies provide a comprehensive suite of counterfactual diagnostics. A reliable causal estimation method should demonstrate stability across bootstrap samples, insensitivity to irrelevant variables, robustness to subsampling, and null effects under placebo or dummy variable conditions.

## Supplementary information

Supplementary Information

## Data Availability

The datasets analyzed in the current study are not publicly available due to patient privacy purposes, but are available upon reasonable request to the corresponding author Shi-Ming Li (lishiming81@163.com).

## Code availability

The code can be made available upon reasonable request to the corresponding author Xu Yang (pyro_yangxu@bit.edu.cn).
