# Supplementary material for: Robust and interpretable unit level causal inference in neural networks for pediatric myopia
Source: NPJ Digit Med. 2026 Feb 19;9:263. doi: 10.1038/s41746-026-02442-7 (PMC13032011; doi:10.1038/s41746-026-02442-7)
Supplement: Supplementary file 1 — Supplementary Information [file 41746_2026_2442_MOESM1_ESM.pdf]

Supplementary material

Robust and Interpretable Unit Level Causal Inference in Neural Networks for Pediatric Myopia

Zihui Jin, Mengtian Kang, Wuyan Zhao, Wenjin Gui, He Li, Yongfang Tu, Yongjun Huo, Canqing Yu, Weihua Song, Ningli Wang, Xu Yang, Shi-Ming Li

---

This PDF file includes:

Supplementary Text

Supplementary Figures (Figures S1-7)

1. Supplementary Text

1.1. Dataset Processing and Feature Construction

To improve data quality and preserve temporal structure, we conducted a three-step preprocessing procedure, including missing value imputation, temporal feature compression, and feature selection.

Missing values were handled differently for variable CR and other variables. For Non-CR data, conventional imputation strategies such as median, mean, or mode were applied. For CR, which is critical to vision development but partially missing in follow-ups, we adopted a stepwise gradient boosting regression (GBRT) strategy. Imputation proceeded iteratively: values for grade 2 were estimated using baseline data, then grade 3 using grades 1–2, and so on, to maintain longitudinal coherence.

Rather than treating the dataset as cross-sectional, we extracted time-aware features to capture long-term patterns. Variables were processed as follows: CR: For each subject, annual CR progression (difference between consecutive years) was calculated. The average of these five progression

values was used as a temporal indicator of myopia development. NW, DTO, CB, EGG, REDM and WHIM: These behavior and diet-related categorical variables were aggregated by summing their values across the six-year period, highlighting cumulative lifestyle exposure. NAR, DAR, PULSE, AL, K1 and K2: As stable physiological indicators, these continuous variables were averaged over the six years. PWG and GENDER: As invariant baseline variables, their values were taken directly from the initial survey.

We employed a combination of univariate filtering and multivariate model-based selection to identify the most informative predictors for myopia classification. Sixteen variables were ultimately selected and used as inputs for model training and causal attribution.

1.2. Causal Learning Basics

Causal Discovery. Causal discovery, also referred to as causal structure learning, is a foundational technique in statistics and machine learning that aims to uncover directed relationships among variables from observational data. In contrast to mere correlation, causal discovery reveals mechanisms—how one variable may induce changes in another—forming the basis for prediction, intervention, and system understanding.

Although randomized controlled trials (RCTs) remain the gold standard for causal inference, their application is often constrained by ethical, logistical, or financial limitations, especially in medical settings. As a result, recent developments increasingly focus on identifying causal structures from purely observational data. Causal discovery algorithms allow researchers to reconstruct causal graphs—typically represented as directed acyclic graphs (DAGs)—based on conditional independence tests among variables.

One key concept in this framework is d-separation, which provides a formal criterion to determine whether a set of variables renders others conditionally independent within a DAG. This enables the systematic elimination of spurious dependencies and supports reliable causal inference even in high-dimensional settings.

Causal Inference. Causal inference is the process of evaluating causal effects, aiming to quantify how a variable causally influences another based on a learned causal graph. The magnitude of such influence is known as the causal effect.

Accurate estimation of causal effects is essential for interpretability and decision-making. Without a reliable understanding of cause-effect relationships, it would be difficult to determine which interventions or policies are effective. For example, identifying the causal effect of a drug on disease recovery enables the selection of the most effective treatment. Although causal inference from observational data is challenging due to confounding and counterfactual unobservability, modern frameworks such as *Structural Causal Models (SCM)* and the *Potential Outcome Framework (POF)* have enabled more reliable estimation of causal effects from data.

Causal inference relies on several core concepts, often defined differently in SCM and POF. Below we provide the general definitions:

- **Unit:** The atomic subject of analysis (e.g., a single patient in a clinical trial).
- **Treatment (or Intervention):** An action applied to a unit (e.g., taking a drug).
- **Outcome:** The result of the treatment, which may be classified as:
  - *Potential Outcome* $Y(t)$: The outcome that would occur under treatment t.
  - *Observed Outcome*: The actual measured result for a unit.
  - *Counterfactual Outcome*: The potential outcomes for treatments not received.

Causal effects can be defined at different levels. Assuming binary treatment $T \in \{0, 1\}$, we have:

- **Average Treatment Effect (ATE):**

$$\text{ATE} = \mathbb{E}[Y(1)] - \mathbb{E}[Y(0)]$$

- **Average Treatment Effect on the Treated (ATT):**

$$\text{ATT} = \mathbb{E}[Y(1) - Y(0) \mid T = 1]$$

- **Conditional Average Treatment Effect (CATE):**

$$\text{CATE}(X) = \mathbb{E}[Y(1) - Y(0) \mid X]$$

- **Individual Treatment Effect (ITE):**

$$\text{ITE}_i = Y_i(1) - Y_i(0)$$

Two Types of Causal Models. SCM is a graph-based framework proposed by Pearl, representing variables and their causal dependencies via directed acyclic graphs (DAGs). An SCM is defined as a triplet $\mathcal{M} = \langle U, V, F \rangle$ where:

- U: A set of exogenous variables (unobserved, external factors),
- V: A set of endogenous variables (internal, observable),
- F: A set of structural equations such that each $V_i = f_i(PA_i, U_i)$, with PA_i being the parents of V_i.

This model enables formal reasoning about interventions using the do-operator, making it possible to simulate counterfactual scenarios and compute causal effects.

Also known as the Rubin Causal Model, the POF frames causal inference around the comparison of counterfactual outcomes. Each unit has two

potential outcomes—only one of which can be observed—while the other is counterfactual.

The model relies on the following key assumptions:

- **Stable Unit Treatment Value Assumption (SUTVA)**: The potential outcome for one unit is unaffected by the treatment assignments of other units.
- **Ignorability:**
$$Y(0), Y(1) \perp T \mid X$$
- **Positivity:** Every unit has a non-zero probability of receiving each treatment, i.e., $\mathbb{P}(T = t \mid X = x) > 0$ for all t, x.

Under these assumptions, POF allows for unbiased estimation of causal effects even from non-experimental data.

1.3. Comparison Across Causal Estimators

We compare our domain-adaptive meta-learning algorithm against several benchmark estimators, including LinearDML, DRLearner, SLearner, XLearner, and TLearner. The results are shown in Table S1.

Table S1: Rscore Comparison Across Causal Estimation Methods

Method	Rscore
LinearDML	0.0205
DRLearner	-0.7904
SLearner	0.0259
XLearner	-0.0127
TLearner	0.1590
DomainAdaptationLearner (Ours)	0.1651

As can be seen from Table S1, the algorithm we proposed in this paper scores higher in Rscore and has better algorithm effect.

1.4. Base Learner Selection for Meta-Model

To further enhance performance, we experimented with various base learners in our meta-learning framework, including Extremely Randomized Trees (ExtraTree), Support Vector Machines (SVM), Random Forest, and Gradient Boosting Decision Trees (GBDT). Table S2 shows the results.

Table S2: Rscore Comparison Across Base Learners

Base Learner	Rscore
ExtraTree	-0.0539
SVM	-0.0369
Random Forest	0.2260
GBDT	0.3850

As can be seen from Table S2, GBDT has the highest score in Rscore, so subsequent experiments will consider using GBDT as the prediction model.

1.5. Ensemble Strategy Using Weighted Voting

We adopted an ensemble strategy combining GBDT and Random Forest via *weighted voting*. As shown in Table S3, we varied the GBDT : Random-Forest weight ratio and recorded the corresponding Rscore. The optimal ratio of 10 : 1 yielded the highest Rscore of 0.6277, indicating superior attribution performance.

Table S3: Comparison of Rscore under Different Weight Settings

GBDT Weight	RandomForest Weight	Rscore
1	1	0.5214
2	1	0.5772
3	1	0.6093
5	1	0.6238
8	1	0.6188
10	1	0.6277
1	2	0.4460
1	3	0.3981
1	5	0.3478
1	8	0.3054
1	10	0.2931

This improvement demonstrates that carefully tuning ensemble weights can significantly boost meta-model accuracy in estimating heterogeneous treatment effects.

2. Supplementary Figures

2.1. Isolated Units Attribution Experiment Results

The attribution experiment results of Isolated Units for NW, DTO and WHIM are shown in Figure S1, S2, S3.

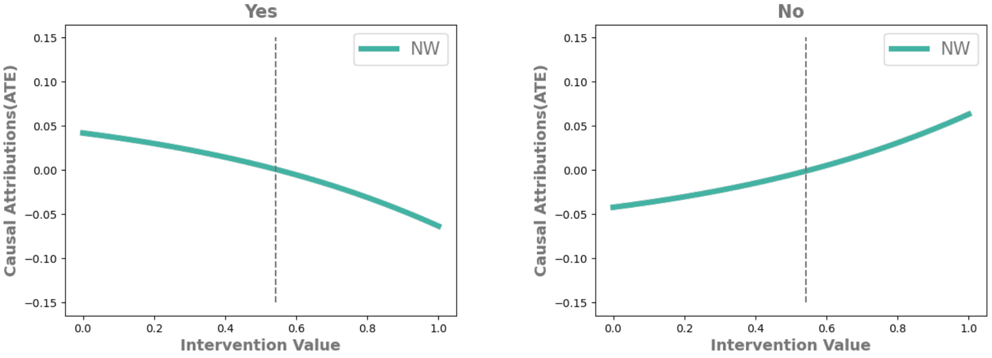

Figure S1: **Causal attribution results of isolated units for NW (total nearwork load)** The difference between the ATE value and its average value is presented as the green line, which shows the increasing/decreasing trend of variable for myopic. 'YES' means myopic and 'NO' means non-myopic. The vertical dotted line indicates that the ATE at this location is 0.

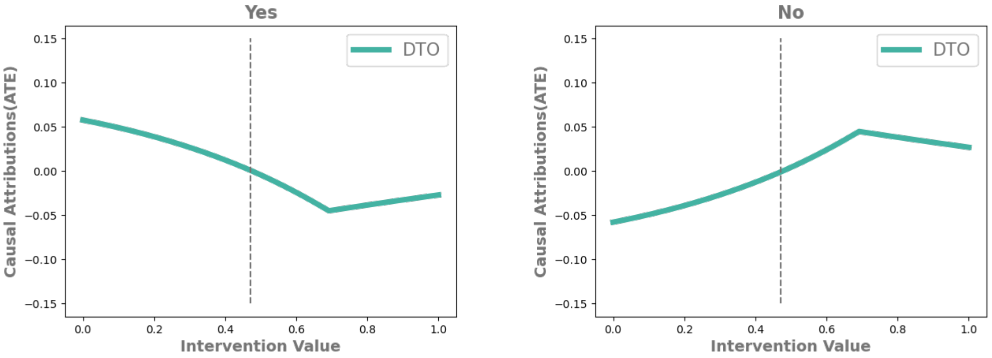

Figure S2: **Causal attribution results of isolated units for DTO (distance-viewing time outdoors)** The difference between the ATE value and its average value is presented as the green line, which shows the increasing/decreasing trend of variable for myopic. 'YES' means myopic and 'NO' means non-myopic. The vertical dotted line indicates that the ATE at this location is 0.

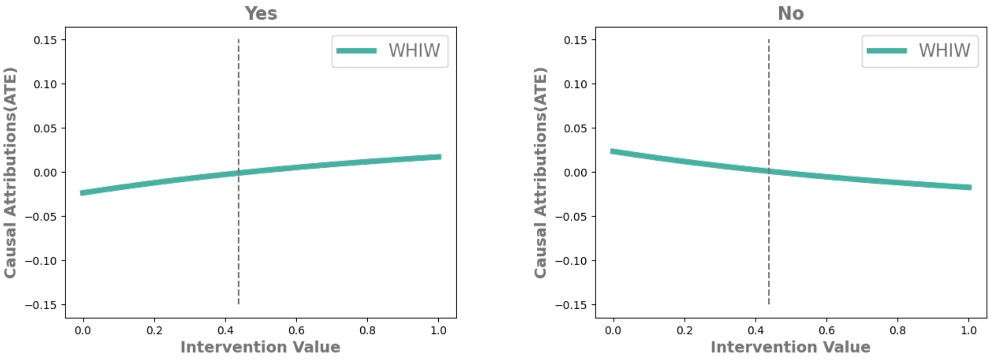

Figure S3: **Causal attribution results of isolated units for WHIM (white meat consumption frequency)** The difference between the ATE value and its average value is presented as the green line, which shows the increasing/decreasing trend of variable for myopic. 'YES' means myopic and 'NO' means non-myopic. The vertical dotted line indicates that the ATE at this location is 0.

2.2. Pure Units Attribution Experiment Results

The attribution experiment results of Pure Units for NAR, DAR, K1 and K2 are shown in Figure S4, S5, S6, S7.

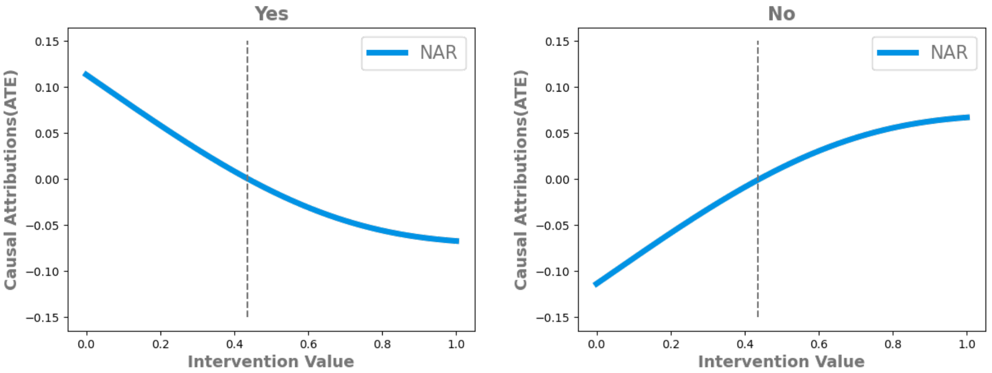

Figure S4: **Causal attribution results of pure units for NAR (near accommodative ability of right eye)** The difference between the ATE value and its average value is presented as the green line, which shows the increasing/decreasing trend of variable for myopic. 'YES' means myopic and 'NO' means non-myopic. The vertical dotted line indicates that the ATE at this location is 0.

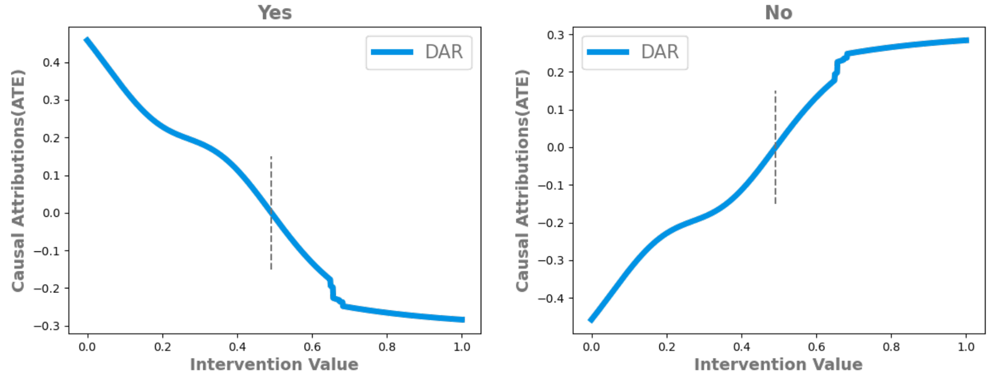

Figure S5: **Causal attribution results of pure units for DAR (distance accommodative ability of right eye)** The difference between the ATE value and its average value is presented as the green line, which shows the increasing/decreasing trend of variable for myopic. 'YES' means myopic and 'NO' means non-myopic. The vertical dotted line indicates that the ATE at this location is 0.

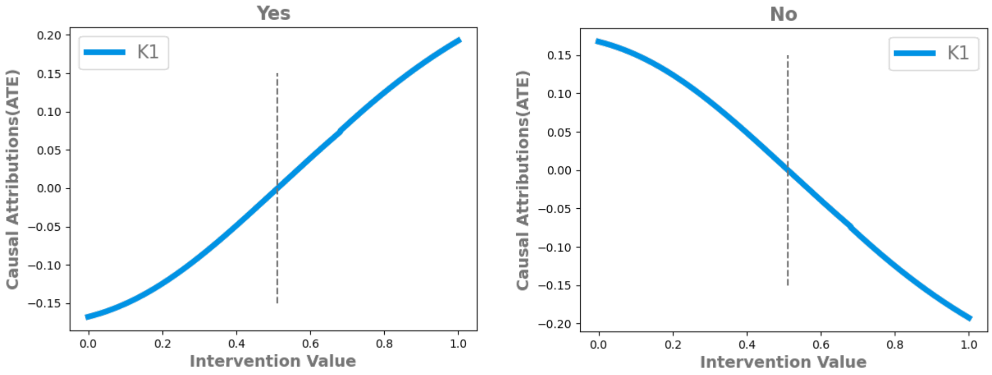

Figure S6: **Causal attribution results of pure units for K1 (corneal keratometry)** The difference between the ATE value and its average value is presented as the green line, which shows the increasing/decreasing trend of variable on myopic. 'YES' means myopic and 'NO' means non-myopic. The vertical dotted line indicates that the ATE at this location is 0.

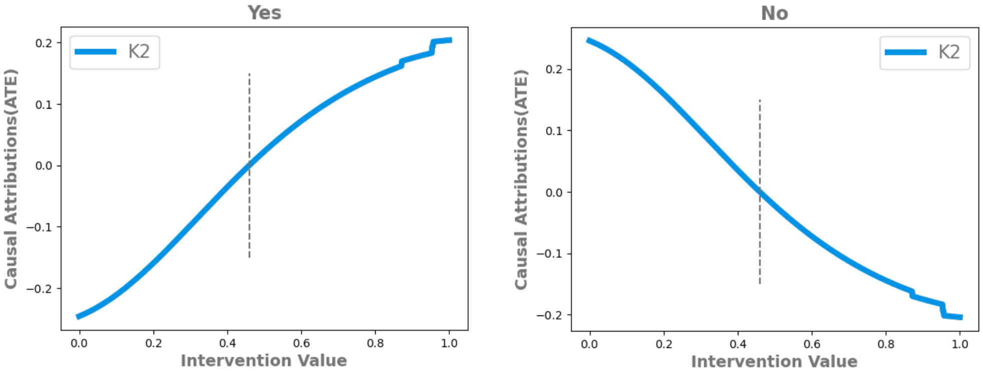

Figure S7: **Causal attribution results of pure units for K2 (corneal keratometry)** The difference between the ATE value and its average value is presented as the green line, which shows the increasing/decreasing trend of variable on myopic. 'YES' means myopic and 'NO' means non-myopic. The vertical dotted line indicates that the ATE at this location is 0.
